# Long-Term Outcome of Endoscopic Balloon Dilation in Obstructive Gastrointestinal Crohn's Disease: A Prospective Long-Term Study

**DOI:** 10.1155/DTE.6.67

**Published:** 2000

**Authors:** Toshiyuki Matsui, Keisuke Ikeda, Sumio Tsuda, Kenshi Yao, Suketo Sou, Shigeru Satoh, Sadamune Hatakeyama, Hiroaki Matake, Toshihiro Sakurai, Tsuneyoshi Yao

**Affiliations:** Department of Gastroenterology Fukuoka University Chikushi Hospital Zokumyouin 377-1, Chikushino Fukuoka 818-8502 Japan

## Abstract

*Background* The short- and long-term results of balloon dilation therapy in Crohn's patients with non-anastomotic obstructive gastrointestinal lesions are investigated.

*Materials and methods* Fifty-five patients with Crohn's disease who had obstructive gastrointestinal lesions were treated prospectively by endoscopic balloon dilation.

*Short-term results* Eight of the initial dilations were unsuccessful giving no symptomatic relief (14.5%).

*Long-term results* The subjects of the long-term prognosis were 40 cases followed up for more than 6 months (average 37 months) and their strictures were non-anastomotic in more than half (59%). Avoidance of surgery, was possible in 31 of 40 patients (78%). Surgery was avoided in 92%, 81% and 77% of patients after one, two, and three years, respectively (Kaplan–Meier's method). There was no difference in long-term outcome between anastomotic strictures and strictures in the absence of prior surgery.

*Conclusion* Our results suggest that, (1) strictures in the absence of prior surgery might be treated in this way as well as anastomotic strictures; (2) if followed for a prolonged time period, more than 70% of patients, who have undergone balloon dilation for obstructive gastrointestinal Crohn's disease, may be able to avoid surgery.


*Diagnostic and Therapeutic Endoscopy*, Vol. 6, pp. 67–75
Reprints available directly from the publisher
Photocopying permitted by license only

© 2000 OPA (Overseas Publishers Association) N.V.
Published by license under
the Harwood Academic Publishers imprint,
part of The Gordon and Breach Publishing Group.
Printed in Malaysia.

### Long-Term Outcome of Endoscopic Balloon Dilation in Obstructive Gastrointestinal Crohn's Disease: A Prospective Long-Term Study

TOSHIYUKI MATSUI*, KEISUKE IKEDA, SUMIO TSUDA, KENSHI YAO, SUKETO SOU, SHIGERU SATOH, SADAMUNE HATAKEYAMA, HIROAKI MATAKE, TOSHIHIRO SAKURAI and TSUNEYOSHI YAO

*Department of Gastroenterology, Fukuoka University Chikushi Hospital, Zokumyouin 377-1, Chikushino, Fukuoka, 818-8502, Japan*

*(Received 19 July 1999; Revised 23 August 1999; In final form 27 August 1999)*

***Background*** **The short- and long-term results of balloon dilation therapy in Crohn's patients with non-anastomotic obstructive gastrointestinal lesions are investigated.**

***Materials and methods*** **Fifty-five patients with Crohn's disease who had obstructive gastrointestinal lesions were treated prospectively by endoscopic balloon dilation.**

***Short-term results*** **Eight of the initial dilations were unsuccessful giving no symptomatic relief (14.5%).**

***Long-term results*** **The subjects of the long-term prognosis were 40 cases followed up for more than 6 months (average 37 months) and their strictures were non-anastomotic in more than half (59%). Avoidance of surgery, was possible in 31 of 40 patients (78%). Surgery was avoided in 92%, 81% and 77% of patients after one, two, and three years, respectively (Kaplan–Meier's method). There was no difference in long-term outcome between anastomotic strictures and strictures in the absence of prior surgery.**

***Conclusion*** **Our results suggest that, (1) strictures in the absence of prior surgery might be treated in this way as well as anastomotic strictures; (2) if followed for a prolonged time period, more than 70% of patients, who have undergone balloon dilation for obstructive gastrointestinal Crohn's disease, may be able to avoid surgery.**

*Keywords:* Balloon dilation, Crohn's disease, Gastrointestinal stricture, Long-term prognosis

### INTRODUCTION

Gastrointestinal stricturing involvement with Crohn's disease is the most common complication requiring surgical intervention, and is occurring at an increasingly high rate. Patients typically present with symptoms of upper and lower intestinal obstruction. In these patients, usual medical therapy has little effect in curing obstruction. Operative resection or strictureplasty are the accepted forms

* Corresponding author. Tel.: +81-92-921-1011. Fax: +81-92-921-0856. E-mail: ff036711@csat.fukuoka-u.ac.jp.

of treatment in segmental stenosis. However, long-term surgical results indicate a high rate of re-operation [1–4].

Endoscopic balloon dilation has been well documented for benign gastrointestinal stenotic lesions [5–23], however, there are few reports concerning Crohn's stenosis [24–33]. Moreover, there is only one study which reported the long-term success rates or complications of endoscopic dilation [30]. Firm conclusions or recommendations have not been drawn concerning balloon dilation because there have been few large long-term studies on the results of balloon dilation in Crohn's patients with obstructive gastrointestinal lesions [27,30]. The aim of the present study is to clarify the long-term efficacy and results of balloon dilation in forty-five patients prospectively, especially in patients with no prior surgery. Long-term prognosis and the factors influencing results were also investigated clinically and radiographically.

### PATIENTS AND METHODS

From 1989 to 1999, 276 patients with Crohn's disease were treated in our department and 55 (19.9%) of these underwent balloon dilation in our institute. All the procedures were performed by study members (T.M., S.T., K.Y. and S.S.) in a uniform procedure.

### Patients

Thirty-two of the 55 patients were male. Age at the onset of gastrointestinal symptoms was ranged from 15 to 36 years (average 26.6). The diagnosis of Crohn's disease was confirmed endoscopically, radiographically or histologically. Before obstructive symptoms occurred, their disease had been followed for an average of 7.9 years (range 0.8–10.0). They were classified into 14 small intestinal (6 gastroduodenal), 34 ileocolitis, and 7 colitis type cases. At the time of dilation 32 (58%) had previously undergone surgery. Six patients had gastroduodenal stenoses and 49 patients had ileocolic stenoses. At the time of dilation 45 patients had mild to severe obstructive symptoms and 10 did not have obvious obstructive symptoms but had severe strictures which an endoscope was not able to pass through. These 10 patients had been recieving nutritional therapy (total parenteral nutrition or elemental diet) for active diseases for several weeks and were on nil by mouth. After disease activity had settled down to remission stage (CDAI $<150$), colonoscopic evaluation revealed severe stenosis in these patients. They did not have obstructive symptoms because they had not ingest an ordinary diet yet. Seven of the ten asymptomatic patients turned out to be symptomatic during follow-up when on a regular diet.

### Indications for Dilation

Indications for balloon dilation were as follows: (1) patients who had strictures in gastroduodenal lesions or ileocolonic lesions, (2) the activity of the disease was quiescent (CDAI $<150$, in 48) or not highly active ($150<\mathrm{CDAI}<250$, in 7), (3) their general condition was not extremely poor and severe complications (abscess, severe fistula, deep ulcers) were lacking. However, patients with occult fistula or fine fistula were included, (4) patients agreed to the procedure. Informed consent was obtained from all patients. Most of the patients had obstructive symptoms, however, asymptomatic patients who had severe stenosis under nutritional therapy were thought to be indicated for this therapy.

Morphological characteristic of lesions in the 55 patients were recorded mainly by pre- and post-dilation radiography using location, length, angulation, with or without oral luminal dilatation, waist size of balloon at maximal dilation, anastomotic or non-anastomotic and concomitant fistula as measures.

### Balloon Dilation Technique

Balloon dilation was performed using 10 and 20 mm balloons (Rigiflex, Microvasive, Milford, Mass.), through a GIF-XQ-30 endoscope for upper-gastrointestinal and a CF-200I endoscope

(both Olympus Optical Company, Tokyo, Japan) for lower intestinal stenoses as noted before [33]. Diazepam was used to sedate patients. Our endoscopic approach was to start with a small through-the-scope (TTS) balloon, then proceed to a larger size and finally to use a large over-the-wire (OTW) balloon, with the progression based on the radiographic and clinical responses of the patients to the dilation. Each dilation session consisted of two to four 5 min inflations of each balloon size used. Under fluoroscopic guidance, the balloon or the guide wire was advanced as far as possible through the stricture and into the distal side. When using an OTW balloon, the endoscope was removed and the balloon catheter was advanced over the guide wire through the stricture. We performed maximal dilation over a few days (separate dilation two or three times) by a way of progressively stretching the strictured lesions. At the initial dilation a small balloon was used with a larger one at the second dilation. Finally, we targeted a maximum diameter of 2 mm in all stenoses under a pressure of control between 20 and 30 pounds per square inch.

Post-dilation studies were obtained using diluted barium whenever possible. The success of the dilation was judged strictly on the basis of symptomatic relief. Some of the patients have been prescribed drugs (mesalamine or corticosteriods) and most of the patients were prescribed an enteral nutritional diet before and after the dilation without significant therapeutic changes. Other food restrictions were not enforced during the follow-up.

### Follow-Up and Re-Dilation

During the follow-up period careful observations were made concerning symptomatic recurrence. After the first dilation session the 40 patients were followed up for 7–95 months (average 37.0). When obstructive symptoms recurred re-dilations were performed as soon as possible in the outpatient clinic or after re-admission. After re-dilation patients were regularly checked and received re-dilation when neccesary in the outpatient clinic.

### Analysis of Short-Term and Long-Term Prognosis

When the first endoscopic dilation was unsuccessful because of severe, long or highly angulated stenoses, patients were only analyzed in the short-term prognosis and were not included as subjects of the long-term analysis. Patients whose follow-up period was less than 6 months were also not included in this study. Forty patients were thus analyzed for long-term prognosis (Fig. 1). Profiles of these are listed

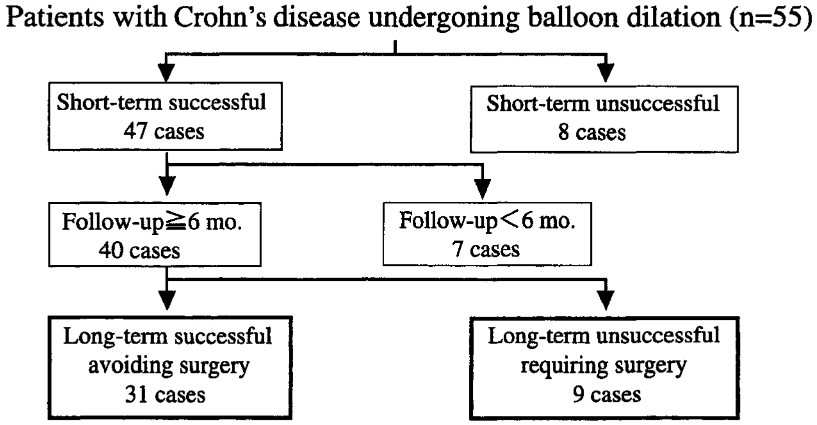

FIGURE 1 Subjects of the study. Fifty-five patients underwent balloon dilation therapy. Short-term success was attained in 47 patients. Forty patients who were followed up for more than 6 months (average 37.0 months) were the subjects for long-term prognosis. Finally 9 patients (22.5%) were operated on.

in Table I. They were classified into 11 small intestinal (6 gastroduodenal), 23 ileocolitis, and 6 colitis type cases, and at the time of dilation 17 (42.5%) had previously undergone surgery. Patients had 6 gastroduodenal, 6 rectal, 13 colonic, 11 ileocecal, and 4 ileal stenoses. At the time of dilation 30 patients had mild to severe obstructive symptoms and 10 did not have obvious obstructive symptoms.

The cumulative avoidance of surgery rate was calculated by the Kaplan–Meier's method.

Comparisons between patients who avoided surgery (successful) and patients who did not avoid surgery (unsuccessful) were made in respect to background and morphological features. Background features compared were: gender, age at dilation, duration of disease, disease type, site of stricture (upper or lower gastrointestinal tract), and patients with or without symptoms.

Morphological and clinical characteristics of the lesions were compared between the two groups as follows: length, with or without angulation, lesion with or without oral luminal dilatation, waist diameter on the balloon by radiography, anastomotic or non-anastomotic, the presence of concomitant fistula and having recurrence or not. The rate of complication was also calculated.

### Statistics

Chi-square test or Fisher's exact test were used when comparing frequency. Student's $t$-test was used when comparing mean values. When $p$ value was less than 0.05 the difference was considered significant.

### RESULTS

*1 Short-term prognosis* Eight cases were unsuccessful at the first dilation and so the short-term success rate was 85.5% (Fig. 1). A stricture longer than 3 cm was the main cause of failure. Extremely angulated or edematous lesions were also not able to be dilated successfully. In one patient with colonic stenosis, peritonitis with sealed perforation occurred and surgical intevention was necessary.

*2 Long-term prognosis in relation to background and morphological characteristics* Background features of the successful and unsuccessful cases were compared. There were no differences in gender, age at dilation, duration of disease, disease type, site of stricture (upper or lower gastrointestinal tract), and the rate of symptomatic cases (Table II). When comparing the morphological

TABLE I Profiles of patients followed for long-time periods after successful dilation

| | |
|---|---|
| Age (years) | 30.1 ± 8.1 |
| Sex (M/F) | 37/13 |
| Duration of disease (years) | 9.1 ± 5.3 |
| Type of disease | |
| S | 11 |
| SL | 23 |
| L | 6 |
| Site of stricture | |
| Gastroduodenal | 6 (0) |
| Rectal | 6 (5) |
| Colonic | 13 (7) |
| Ileocecal | 11 (4) |
| Ileal | 4 (1) |
| Total | 40 (17) |
| Concomitant fistula | 8 |

( ): number of anastomotic strictures, S: small intestinal, SL: small and large intestinal, L: large intestinal.

TABLE II Background features of patients with Crohn's disease undergoing balloon dilation

| Backgrounds | Successful cases $n = 31$ | Unsuccessful cases $n = 9$ |
|---|---|---|
| M/F | 21/10 | 6/3 |
| Age (years) | 30.1 ± 7.2 | 29.6 ± 9.0 |
| Duration of disease (years) | 8.4 ± 5.0 | 8.8 ± 4.7 |
| Type of disease | | |
| S | 9 | 2 |
| SL | 18 | 5 |
| L | 4 | 2 |
| Site of stricture | | |
| Upper | 4 | 2 |
| Lower | 27 | 7 |
| Symptom | | |
| + | 23 | 6 |
| – | 8 | 3 |

S: small intestine, L: large intestine.

TABLE III Morphological and clinical characteristics of patients with Crohn's disease undergoing baloon dilation

| Characteristics | Successful cases $n = 31$ | Unsuccessful cases $n = 9$ |
|---|---|---|
| Length (mm) | 20.8 ± 10.8 | 17.0 ± 6.0 |
| Angulation | | |
| + | 14 | 6 |
| − | 17 | 3 |
| Oral dilatation | | |
| + | 9 | 5 |
| − | 22 | 4 |
| Waist diameter (mm) | 15.3 ± 3.9 | 17.8 ± 3.0 |
| Anastomotic | 12 | 5 |
| Non-anastomotic | 19 | 4 |
| Concomitant fistula | | |
| + | 5 | 3 |
| − | 26 | 6 |
| Symptomatic recurrence* | | |
| + | 14 | 8 |
| − | 17 | 1 |

*< 0.05.

characteristics of the lesions between the two groups, there were no significant differences in length, with or without angulation, waist diameter on the balloon, anastomotic or non-anastomotic, presence of concomitant fistula and with or without oral luminal dilatation. However, patients with recurrent symptoms had a significantly higher failure rate ($p < 0.05$, Table III).

*3 Re-dilation* Symptomatic recurrences occurred in 22 patients (55%) during the follow-up period and all these patients received re-dilation. Ten patients (25%) had more than two recurrences. Six of these received scheduled dilation every 3–9 months. One hundred and fifteen dilations (range: 1–8 times/person) were performed in the 40 patients.

*4 Long-term avoidance of surgery rate* (Fig. 2) Seven patients were operated on because of frequent recurrences or for intractable fistulas, one because of a perforation after the second dilation and one because of massive bleeding unrelated to the dilation procedures (Table IV). From the analysis of the 40 patients, avoidance of surgery was possible in 31 (77.5%) and serial rates were calculated as 92% in one, 81% in two, 77% in three and 68% in five years (Fig. 2).

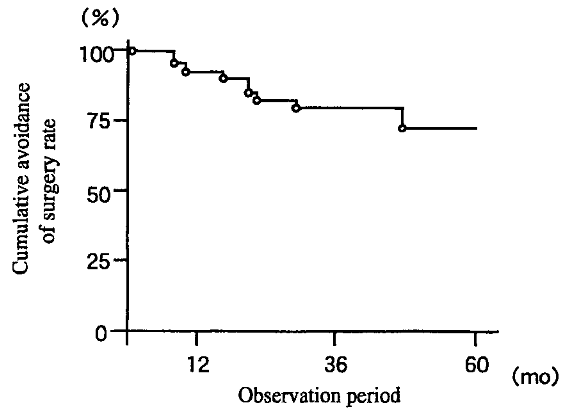

FIGURE 2 The cumulative avoidance of surgery rate after balloon dilation in patients with Crohn's disease (Kaplan–Meier's method).

### DISCUSSION

Hydrostatic balloons have been used for dilation of gastrointestinal strictures of diverse etiology [5–33]. Balloon dilation has been successfully performed for Crohn's patients with gastrointestinal obstructive lesions [24–33]. Although there have been some studies which have examined the efficacy of treatment of obstructive ileocolonic Crohn's disease by balloon dilation there are very few long-term studies in which dilations were prospectively performed with a large number of cases [27,30]. Couckuyt reported that the long-term prognosis of a large number of patients who received balloon dilation was fairly good (at a mean follow-up of 34 months, 34 (62%) patients remained unoperated on) but there was a relatively high complication rate (11%) [30]. However, most of the reported Crohn's patients included in Couckuyt's series had anastomotic strictures and endoscopic balloon dialtion for anastomotic strictures in Crohn's disease has been considered to be a first-choice modality of treatment. However, there were no conclusions whether non-anastomotic strictures in Crohn's disease were indicated for balloon dilation or not. Moreover, factors influencing the long-term results were not fully analyzed. Our previous results, with non-anastomotic gastroduodenal obstructive lesions in Crohn's disease, were satisfactory because

TABLE IV Crohn's patients requiring surgery after balloon dilation

| No. | Age/sex | Location | Symptom | Lesion characteristic | | | | Observation period (months) | Reasons for surgery |
|---|---|---|---|---|---|---|---|---|---|
| | | | | Length (mm) | Angulation | Oral dilatation | Waist diameter (mm) | | |
| 1 | 34F | Post-bulbar | + | 30 | + | − | 14 | 8 | Frequent recurrence |
| 2 | 22M | Post-bulbar | + | 12 | + | − | 17 | 21 | Frequent recurrence |
| 3 | 24M | Ileorectal anastomosis | + | 22 | + | + | 10 | 21 | Recurrence intractable fistula |
| 4 | 34F | Ileorectal anastomosis | + | 10 | − | + | 17 | 32 | Intractable fistula |
| 5 | 31M | Ileorectal anastomosis | + | 30 | + | + | 18 | 21 | Frequent recurrence intractable fistula |
| 6 | 25M | Ileoascending anastomosis | − | 15 | − | + | 14 | 10 | Frequent recurrence |
| 7 | 32M | Transversoileal anastomosis | + | 11 | − | + | 17 | 50 | Frequent recurrence |
| 8 | 22F | Ileocecal | − | 15 | − | − | 16 | 24 | Bleeding |
| 9 | 29M | Transverse colon | − | 15 | − | − | 14 | 8 | Peritonitis after the 2nd dilation |

of the fairly good long-term patency of the lesion although high rates of recurrence were observed [33]. Although different endoscopic approaches were necessary for gastroduodenal and the ileocolic stenoses, there were no obvious differences in dilation procedure, long-term success rate and the rates of recurrences between the two sites of the stenoses. Including these cases, short and long-term outcomes and the affecting factors were analyzed prospectively, especially in the non-anastomotic patients in this study.

Initial dilations were successful in 85.5% of patients in the present study. From a technical point of view, balloon dilation might not produce favorable results in extremely long or angulated lesions, as our results show and other reports have shown [27,29,31,33].

As for long-term outcomes, our results suggested that if followed for a prolonged period of time, patients who have undergone balloon dilation for obstructive gastrointestinal Crohn's lesions have high (55%) rates of symptomatic obstruction recurrences. However, re-dilations were successful in most of the patients, which then resulted in an avoidance of surgery. Therefore, in carefully selected Crohn's patients, balloon dilation could be considered beneficial in combination with other medical treatments.

Obviously, the balloon dilation technique has been considered to be closely related to the success rate [16,20,24,27,29,30,32]. We have performed maximal dilation over several days by progressive stretching of the strictured lesion. The rapidity with which the desired luminal diameter is achieved is controversial. This slow stretching schedule produced a complication rate lower than other reports [30]. Goldthorn and Recci have proposed that serial dilation may produce the best outcome, by progressively stretching organizing scar tissue [10,20]. If the initial dilation with a small balloon was easily accomplished, the balloon sizes were gradually increased until the desired diameter was achieved. As stated in our previous study, a combination of TTS and OTW balloons has resulted in relatively good outcomes not only in the gastroduodenal strictures, but also in intestinal strictures.

Factors which influence the short- and long-term results were classified according to the patient's background and morphological aspects of the stricture. Several factors were known to effect the outcome of the procedure; anastomotic stricture [9], early recurrence [27], having severe symptoms [27], having deep ulcerations [17], gender [17], long stricture [29,30], angulation [29], high activity [29]. From our results, however, we did not find any significant risk factors other than symptomatic recurrence.

Although the length of the lesion was obviously the most important factor affecting short-term prognosis, the length was not important in respect of long-term prognosis in this study. This because recurrent inflammation at the narrowed area caused symptomatic recurrence with obstruction, fistula formation or massive hemorrhage regardless of the length of the lesion. If the activity of the disease can be controlled with appropriate medical and nutritional therapy, long-term prognosis will usually be more favorable.

There are a few reports that non-anastomotic strictures were effectively dilated by balloon dilation [28–33], and the efficacy of balloon dilation to non-anastomotic strictures (83%) was confirmed as well as that to anastomotic strictures (71%), in our study. This result suggests that when the activity of the patient is low after medical and nutritional therapy, and while the stricturing site does not have deep ulceration, balloon dilation might be indicated to resolve obstructive symptoms.

Less favorable results were obtained in strictures with oral luminal dilatation, which suggested that lesions with longstanding strictures might not dilate easily because of dense and thickened fibrous connective tissue around the strictures.

Significantly, worse results were recognized in patients with recurrent obstructive symptoms and were observed in more than half the patients who underwent dilation. As stated before, recurrence did not result from inadequate dilation technique, nor from other obvious morphological characteristics, but resulted from the natural course of the relapsing disease. Although more than half of the recurrent patients responded to re-dilation, we were not able to find the factors which related to the recurrence. Recurrent patients have to be re-dilated as much as is possible. If recurrence happens again, then re-dilation in an outpatient clinic should be undertaken so long as the response to dilation is sustained. It is speculated that serial dilations be set up in a scheduled fashion so as to preempt symptoms of obstruction from recurring, much like the current practice in chronic esophageal strictures, as Goldthorn reported [10]. However, all patients experienced at least a temporary effect, so that balloon dilation can be considered for use in situations where it is desireable to avoid or postpone surgery.

### *Acknowledgment*

This work was supported by a grant from the Japanese Foundation for Research and Promotion of Endoscopy. The authors are grateful to Mr. Kerry Greer for checking the English of the manuscript.

### *References*

[1] Michelassi, F., Balestracci, T., Chappell, R. *et al.* Primary and recurrent Crohn's disease. *Ann. Surg.* 1991; **214**: 230–238.
[2] Williams, J.G., Wong, W.D., Rothenberger, D.A. *et al.* Recurrence of Crohn's disease after resection. *Br. J. Surg.* 1991; **78**: 10–19.
[3] Stebbing, J., Jewell, D., Kettlewell, M. *et al.* Long-term results of recurrence and reoperaion after strictureplasty for obstructive Crohn's disease. *Br. J. Surg.* 1996; **82**: 1471–1474.
[4] Ozuner, G., Fazio, V.W., Lavery, I.C. *et al.* Reoperative rates for Crohn's disease following strictureplasty. *Dis. Colon. Rect.* 1996; **39**: 1199–1203.
[5] Kozarek, R.A., Hydrostatic balloon dilatation of gastrointestinal stenosis: a national survey. *Gastrointest. Endosc.* 1986; **32**: 15–19.
[6] Hogan, R.B., Hamilton, J.K. and Polter, D.E., Preliminary experience with hydrostatic balloon dilatation of gastric outlet obstruction. *Gastrointest. Endosc.* 1986; **32**: 71–74.
[7] Graham, D.Y., Tabibian, N., Schwartz, J.T. *et al.* Evaluation of the effectiveness of through-the-scope balloons as dilators of benign and malignant gastrointestinal strictures. *Gastrointest. Endosc.* 1987; **33**: 432–435.
[8] McLean, G.K., Cooper, G.S., Hartz, W.H. *et al.* Radilogically guided balloon dilatation of gastrointestinal strictures. Part I. Technique and factors influencing procedural success. *Radiology* 1987; **165**: 35–40.
[9] McLean, G.K., Cooper, G.S., Hartz, W.H. *et al.* Radilogically guided balloon dilatation of gastrointestinal strictures. Part II. Results of long-term follow-up. *Radiology* 1987; **165**: 41–43.
[10] Goldthorn, J.F., Ball Jr., W.S., Willkinson, L.S. *et al.* Esophageal strictures in children: treatment by serial balloon catheter dilation. *Radiology* 1984; **153**: 655–658.
[11] Benjamin, S.B., Glass, R.L., Cattau, E.L. *et al.* Preliminary experience with balloon dilation of the pylorus. *Gastroint. Endosc.* 1984; **30**: 93–95.
[12] Kozarek, R.A., Endoscopic gruntzig balloon dilation of gastrointestinal stenosis. *J. Clin. Gastroenterol.* 1984; **6**: 401–407.
[13] Lindor, K.D., Ott, B.J. and Hughes, R.W., Balloon dilatation of upper digestive tract strictures. *Gastroenterology*; 1985; **89**: 545–548.
[14] Griffin, S.M., Chung, S.C.S., Leung, J.W.C. *et al.* Peptic pyrolic stenosis treated by endoscopic ballon dilation. *Br. J. Surg.* 1989; **76**: 1147–1148.
[15] Schmeudderich, W., Harloff, M. and Riemann, J.F., Through-the-scope balloon dilation of benign pyloric stenosis. *Endoscopy* 1989; **21**: 7–10.
[16] Kozarek, R.A., Botoman, V.A. and Patterson, D.J., Long-term follow-up in patients who have undergone balloon dilatation for gastric outlet obstruction. *Gastrointest. Endosc.* 1990; **36**: 558–561.
[17] Kuwada, S.K. and Alexander, G.L., Long-term outcome of endoscopic dilation of nonmalignant pyloric stenosis. *Gastrointest. Endosc.* 1995; **41**: 15–17.
[18] Misra, S.P. and Dwivedi, M., Long-term follow-up of patients undergoing balloon dilation for benign pyloric stenosis. *Endoscopy* 1996; **28**: 552–554.
[19] Bedogni, G., Rici, E., Pedrazzoli, C. *et al.* Endoscopic dilation of anastomotic colonic stenosis by different technique: an alternative to surgery. *Gastrointest. Endosc.* 1987; **33**: 21–26.
[20] Recci, E., Congliaro, R., Mortella, G. *et al.* Endoscopic management of colonic stenoses. *Endosc. Rev.* 1989; **6**: 9–25.
[21] Murphy, U.K., Repeated hydrostatic balloon dilation in obstructive gastroduodenal Crohn's disease. *Gastrointest. Endosc.* 1991; **37**: 484–485.
[22] Johansson, C., Endoscopic dilation of rectal strictures. A prospective study of 16 cases. *Dis. Colon Rectum* 1996; **39**: 423–428.
[23] Virgillo, C., Cosentino, S., Favara, C. *et al.* Endoscopic treatment of postoperative colonic strictures using an achalasia dilator: short-term and long-term results. *Endoscopy* 1995; **27**: 219–222.
[24] Mohandas, K.M., Swaroop, V.S. and Desai, D.C., Dilation of difficult gastrointestinal strictures using a modified wire-guided technique. *Endoscopy* 1995; **27**: 446–448.
[25] Brower, R.A., Hydrostatic balloon dilation of a terminal ileal stricture secondary to Crohn's disease. *Gastrointest. Endosc.* 1986; **32**: 38–41.
[26] Dobson, H.M. and Robertson, D.R.A., Case-report. Balloon catheter dilation of an ileocolonic stricture. *Clin. Radiol.* 1988; **39**: 202–204.
[27] Blomberg, B., Rolny, P. and Jaerrerot, G., Endoscopic treatment of anastomotic strictures in Crohn's disease. *Endoscopy* 1991; **23**: 195–198.
[28] Williams, A.L. and Palmer, K.R., Endoscopic balloon dilatation as a therapeutic option in the management of intestinal strictures resulting from Crohn's disease. *Br. J. Surg.* 1991; **78**: 453–454.
[29] Junge, U. and Zuechner, H., Endoscopic balloon dilatation of symptomatic strictures in Crohn's disease. *Dtsch. Med. Wschr.* 1994; **119**: 1377–1382.

[30] Couckuyt, H., Gevers, A.M., Coremans, G. *et al.* Efficacy and safety of hydrostatic balloon dilatation of ileocolonic Crohn's strictures: a prospective long-term analysis. *Gut* 1995; **36**: 577–580.

[31] Ramboer, C., Verhamme, M., Dhondt, E. *et al.* Endoscopic treatment of stenosis in recurrent Crohn's disease with balloon dilatation combined with local corticosteroid injection. *Gastrintest. Endosc.* 1995; **42**: 252–255.

[32] Kaila, V.L., El-Newisi, H.M. and Mihas, A.A., Successful endoscopic dilation of a Crohn's colonic stricture. *Gastrointest. Endosc.* 1996; **44**: 359–360.

[33] Matsui, T., Hatakeyama, S., Ikeda, K. *et al.* Long-term outcome of endoscopic balloon dilation in obstructive gastroduodenal Crohn's disease. *Endoscopy* 1997; **29**: 640–645.

